# MiRNA Detection Using a Rolling Circle Amplification and RNA-Cutting Allosteric Deoxyribozyme Dual Signal Amplification Strategy

**DOI:** 10.3390/bios11070222

**Published:** 2021-07-04

**Authors:** Chenxin Fang, Ping Ouyang, Yuxing Yang, Yang Qing, Jialun Han, Wenyan Shang, Yubing Chen, Jie Du

**Affiliations:** State Key Laboratory of Marine Resource Utilization in South China Sea, College of Materials Science and Engineering, Hainan University, Haikou 570228, China; fcx19962021@163.com (C.F.); oypoypoypylyz@163.com (P.O.); thea0208@163.com (Y.Y.); 15859267122@139.com (Y.Q.); jialun_han@126.com (J.H.); 15501862735@163.com (W.S.); robynnchan@foxmail.com (Y.C.)

**Keywords:** signal amplification, deoxyribozyme, rolling-circle amplification, miRNA detection

## Abstract

A microRNA (miRNA) detection platform composed of a rolling circle amplification (RCA) system and an allosteric deoxyribozyme system is proposed, which can detect miRNA-21 rapidly and efficiently. Padlock probe hybridization with the target miRNA is achieved through complementary base pairing and the padlock probe forms a closed circular template under the action of ligase; this circular template results in RCA. In the presence of DNA polymerase, RCA proceeds and a long chain with numerous repeating units is formed. In the presence of single-stranded DNA (H1 and H2), multi-component nucleic acid enzymes (MNAzymes) are formed that have the ability to cleave substrates. Finally, substrates containing fluorescent and quenching groups and magnesium ions are added to the system to activate the MNAzyme and the substrate cleavage reaction, thus achieving fluorescence intensity amplification. The RCA–MNAzyme system has dual signal amplification and presents a sensing platform that demonstrates broad prospects in the analysis and detection of nucleic acids.

## 1. Introduction

The term “nucleic acids” is a general one covering both deoxyribonucleic acid (DNA) and ribonucleic acid (RNA). Nucleic acids are biological macromolecular compounds formed by the polymerization of many nucleotide monomers and form the building blocks of all known forms of life. DNA plays an extremely important role in carrying genetic information, genetic mutations of organisms and the biosynthesis of proteins. RNA plays an important role in protein synthesis. MicroRNA (miRNA) is a class of non-coding, single-stranded RNA with a length of approximately 22 nucleotides encoded for by endogenous genes. miRNAs are involved in post-transcriptional gene expression regulation in animals and plants [1]. In terms of biological mechanisms, miRNA is actively secreted by tumor cells and can act as a tumor marker. As tumor cells form and decay, miRNA expression varies [2]. Therefore, the expression level of each miRNA represents information about human health or disease at a certain time. Numerous miRNAs have been linked to various diseases in humans including cancer [3], human immunodeficiency viruses [4], diabetes [5] and Alzheimer’s disease [6]. Therefore, disease prediction can be achieved via miRNA detection.

There are many different miRNAs in the human body however, they are present in extremely low levels and accurate detection is challenging. Numerous miRNA detection methods have been proposed and traditional methods include Northern blotting [7], microarrays [8] and real-time, fluorescence-based quantitative polymerase chain reaction (RT-PCR) [9]. Although Northern blotting requires relatively simple equipment, the sensitivity and specificity is inadequate. Microarray-based methods usually require separation, labeling and purification of samples prior to hybridization; therefore, the process is complicated and sample purification costs are high. The sensitivity and specificity of RT-PCR methods have been improved and operation is relatively simple; however, the reaction requires precise temperature control and expensive equipment.

In recent years, increasing numbers of miRNA sensor systems have been reported. These new strategies have considerably improved the sensitivity and specificity of miRNA detection. Examples of such new strategies include colorimetric methods [10,11,12], electrochemical detection [13,14,15] and fluorescence-based detection. The advantages of fluorescence detection methods include high detection sensitivity, reduced influence of temperature and improved selectivity. Therefore, fluorescence detection is widely used for the detection of miRNA.

The detection of miRNA is challenging as it is present in low concentrations; therefore, signal amplification strategies are required. Numerous signal amplification strategies have been introduced for miRNA detection such as hybridization chain reaction (HCR) [16,17], catalytic hairpin assembly (CHA) [18,19], strand displacement amplification reaction (SDA) [20,21] and rolling circle amplification reaction (RCA). The method reported herein utilizes RCA.

RCA is an isothermal signal amplification technique. Generally, the reaction is initiated via hybridization of the primer and the padlock probe. Following hybridization, a ligase is added into the system to close the padlock probe into a loop and form a circular template. This short-stranded nucleic acid, which is complementary to the probe, serves as a primer to amplify the DNA reaction. After the polymerase and 2’-deoxynucleotide-5’-triphosphate (dNTPs) are added, the short-stranded nucleic acid is continuously amplified and the formation of a long, single-stranded DNA with repeat sequences is achieved. Due to the formation of several repeating units, RCA achieves the signal amplification effect [22]. Signal amplification using RCA can, in theory, amplify the signal 10^9^ times [23]. Zhuang et al. pioneered the combined use of RCA and CHA to form deoxyribozymes for the detection of miRNAs [24].

Deoxyribozymes are single-stranded DNA fragments with catalytic functions synthesized via in vitro molecular evolution. Joyce et al. reported that a synthetic deoxyribonucleotide catalyzed the cleavage of specific RNA sequences [25]. Compared with traditional RNases, deoxyribozymes have many advantages: (1) Traditional RNases are susceptible to temperature and lose their activity at high or low temperatures, but deoxyribozymes are not affected by temperature. (2) Deoxyribozyme synthesis is relatively simple and low cost. (3) The DNAzyme sensing system has good selectivity and can independently design sequences to catalyze specific units. Therefore, deoxyribozymes offer promise in biosensing applications for the detection of DNA [26], RNA [27] and proteins [28]. Usually, RNA-cutting DNAzymes include a catalytic core region and two substrate binding arms that bind to the substrate through complementary base pairing and in the presence of magnesium ions (Mg^2+^) substrate cleavage occurs. Mokany et al. modified the structure of a previously reported nucleic acid-cleaving DNAzyme to divide the original catalytic core into two parts and added an effector recognition arm. DNAzymes of this structure are called multi-component nucleic acid enzymes (MNAzymes) [29]. The addition of an effector recognition arm results in improved combination of the catalytic core area and effectors, ultimately leading to improved substrate cleavage efficiency. Such MNAzymes have been utilized in biosensor design and as nanomachine components [30,31,32].

In this study, a strategy combining RCA with MNAzymes is presented for the detection of miRNA-21 using dual signal amplification. The design strategy is as follows: miRNA-21 drives the RCA reaction and, once complete, this promotes the formation of MNAzymes. The subsequent addition of Mg^2+^ results in cleavage of the substrate and as the two sides of the substrate contain fluorescent and quenching groups, the fluorescence intensity increases following substrate cleavage.

## 2. Materials and Methods

### 2.1. Reagents and Materials

#### 2.1.1. Materials

Tris buffer solution (1 mol L^−^^1^, pH 8.0), dNTP mixture (25 mmol L^−^^1^) and RNase inhibitor were purchased from Solarbio Life Sciences (China). Sodium chloride and magnesium chloride were purchased from Macklin Biochemical Co., Ltd (Shanghai, China). The microRNA, padlock probe and three oligonucleotides (Table 1) were synthesized and HPLC-purified by Sanggon Biotech Co., Ltd. (Shanghai, China). Ultrapure water was purchased Dongsheng Biotech Co., Ltd. (Guangzhou, China). T4 DNA ligase, phi29 polymerase, exonuclease I (EXO I) and bovine serum albumin (BSA) were purchased from New England Biolabs (Ipswich, MA, USA).

#### 2.1.2. Instrumentation

Fluorescence measurements were performed using a Model RF-6000 fluorescence spectrophotometer (Shimadzu, Japan). A 5’6-fluorescein (FAM) fluorescence dye was used with excitation and emission wavelengths of 494 nm and 520 nm, respectively.

### 2.2. Experimental Procedures

#### 2.2.1. Circular Probe Fabrication

A 2.5 µL padlock probe (20 nmol L^−^^1^) was hybridized with miRNA (an appropriate amount, the dosage of miRNA-21 in the optimized test conditions was 50 pM)by heating at 95 °C for 5 min and then slowly cooling to room temperature for more than 15 min. Next, 1 µL of T4 DNA ligase buffer (10×), 200 U of T4 DNA ligase and 20 U of RNase inhibitor were added to the hybridization system and H_2_O was added to a final volume of 10 µL. The system was held at 37 °C for 2 h and then the reaction was terminated using a temperature of 65 °C for 10 min. Finally, 1 µL of EXO I was added to the solution and the system was held at 37 °C for 1 h, then the reaction was terminated using a temperature of 80 °C for 15 min. Formation of the closed circular probe was then achieved.

#### 2.2.2. RCA Reaction

The RCA reaction system consisted of 11 µL of circular probe (using miRNA as primer to initiate rolling circle amplification reaction and the dosage of miRNA-21 in the optimized test conditions was 50 pM), 2.5 µL of 10× phi29 DNA polymerase buffer, 12.5 U of phi29 DNA polymerase, 5 µL of dNTPs (25 mmol L^−^^1^) and 1 µL of BSA, H_2_O was added to a final volume of 25 µL. The RCA reaction was conducted at 30 °C for 4 h. Finally, the reaction was terminated using a temperature of 65 °C for 10 min.

#### 2.2.3. DNAzyme and Cleavage Reaction

Two single-stranded oligonucleotides (2.5 µL of H1 and 2.5 µL of H2) and NaCl (60 nmol L^−^^1^) were added to the RCA reaction system. The reaction was performed at 95 °C for 5 min and then at room temperature for 2 h. 

Next, the final single-stranded oligonucleotide substrate (2.5 µL) and 15 µL of MgCl_2_ were added to the solution. Then, the cleavage reaction was performed at room temperature for 1.5 h. The product was stored in a refrigerator at 4 °C.

## 3. Results and Discussion

### 3.1. Principle of the microRNA Detection Method

**Figure 1:**
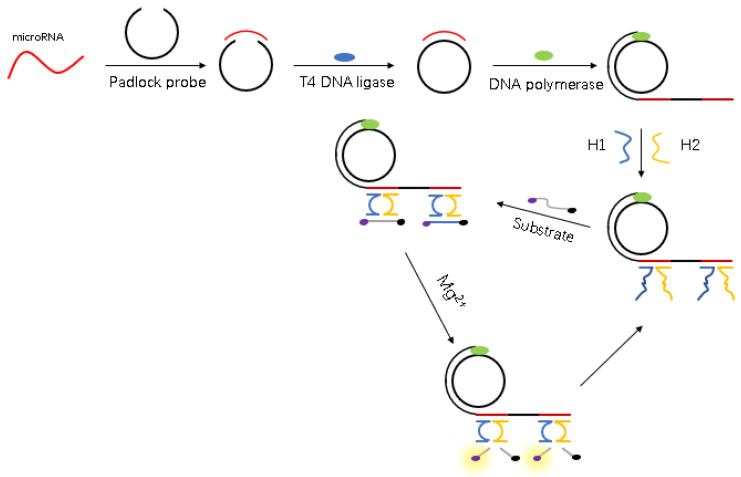
Schematic illustration of the fluorescence assay for the detection of miR-21 using RCA and DNAzyme.

Figure 1 illustrates the design strategy for microRNA detection using Mg^2+^-dependent DNAzyme and RCA. Firstly, the target microRNA hybridizes with the padlock probe via complementary base pairing to achieve a closed loop in the presence of DNA ligase. Secondly, the RCA reaction proceeds when DNA polymerase dNTPs are added into the DNA ligase system to form long-stranded DNA chains with numerous repeating units. Thirdly, when single-stranded DNA (H1 and H2) is added to the polymeric product, hybridization with long-stranded DNA chains forms the DNAzyme. Following the addition of Mg^2+^ and substrate into the DNAzyme system, a catalytic core is formed and the substrate cleavage reaction begins. As the substrate contains fluorophore and quenching groups, the fluorescence intensity substantially increases following cleavage. The substrate simultaneously removes the RCA products and drives the next cleavage reaction. When the target miRNA is mismatched, the properties of T4 DNA ligase mean that formation of a closed loop is challenging: two adjacent and completely complementary DNA/RNA strands must be catalyzed by DNA ligase to form a phosphodiester bond. If the closed loop is absent, the nucleic acid amplification reaction using a circular DNA template does not progress and formation of the DNAzyme does not occur. Following the addition of Mg^2+^, the nucleic acid cleavage reaction does not occur and the fluorescence intensity remains relatively low.

### 3.2. Method Feasibility

During the first stage of the reaction, the concentrations of microRNA and the reactants involved in the ligation reaction are different. The more reactants present, the more generation of circular templates increases. In the second stage, an increased number of cyclic templates are involved in the polymerization reaction generating more long chains. In the third stage, the longer the chains present, the easier DNAzyme formation is; therefore, the substrate cleavage reaction is more likely to occur and an increase in fluorescence intensity follows.

**Figure 2:**
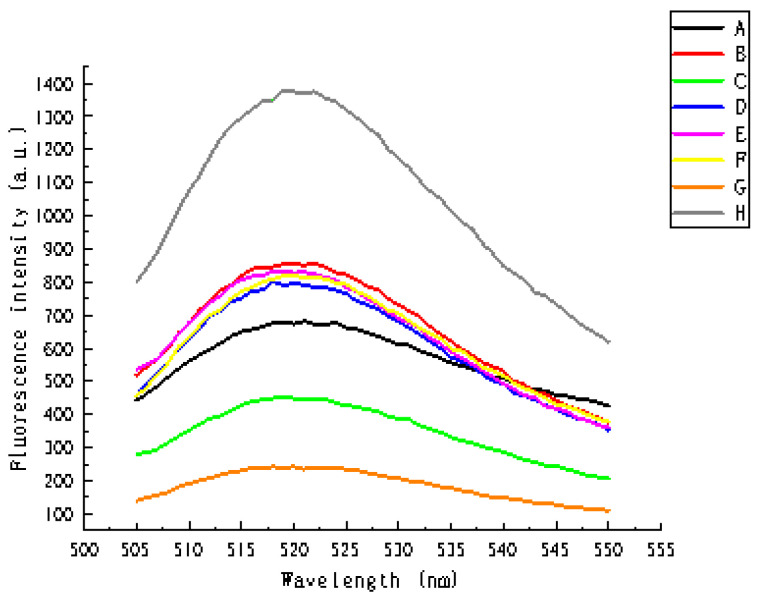
Fluorescence spectra in the absence of sensing system components: (**A**) without miRNA, (**B**) without padlock probes, (**C**) without dNTPs, (**D**) without H1 and H2, (**E**) without Mg^2+^, (**F**) without polymerase, (**G**) without ligase and (**H**) control sample (experimental system in which all the above components exist).

As the reaction in this study results in increased fluorescence intensity, a feasibility analysis was conducted based on fluorescence intensity changes. Figure 2 shows the fluorescence spectra in the absence of sensing system components. It can be seen from the figure that, except for the control group, at 520 nm the fluorescence intensity of any component is relatively low. This confirms the feasibility of the proposed reaction system.

### 3.3. Experimental Parameter Optimization

The subsequent optimization experiment was carried out by changing the concentration of one substance (the concentration of the other substances remains unchanged) on the basis of Section 2.2.

#### 3.3.1. Padlock Probe

**Figure 3:**
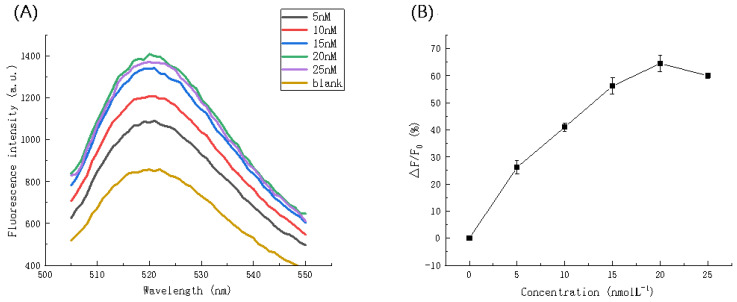
(**A**) Fluorescence spectra and (**B**) Fluorescence intensity ratio (△F/F_0_) at different concentrations of padlock probe.

The padlock probe hybridizes with the target microRNA and becomes an amplification template. Therefore, the concentration of the padlock probe is an important parameter that influences fluorescence intensity. As shown in Figure 3A, the fluorescence intensity increases from 0 nmol L^−^^1^ to 20 nmol L^−^^1^ and is maximal at a concentration of 20 nmol L^−^^1^, all other concentrations result in lower fluorescence intensity values. Figure 3B illustrates the rate of fluorescence intensity change with that observed at a concentration of 20 nmol L^−^^1^ being the highest value (1409.474 a.u.). Therefore, a concentration of 20 nmol L^−^^1^ was selected for the padlock probe.

#### 3.3.2. DNA Ligase

The padlock probe hybridizes with the target RNA via base pairing and the probe then forms a closed loop within the presence of T4 DNA ligase. The ligase catalyzes the formation of phosphodiester bonds between the adjacent 5’-phosphate end and the 3’-hydroxyl end of double-stranded DNA or RNA as well as catalyzing the reaction between blunt and sticky ends. The ligase dosage has a significant influence on fluorescence intensity.

**Figure 4:**
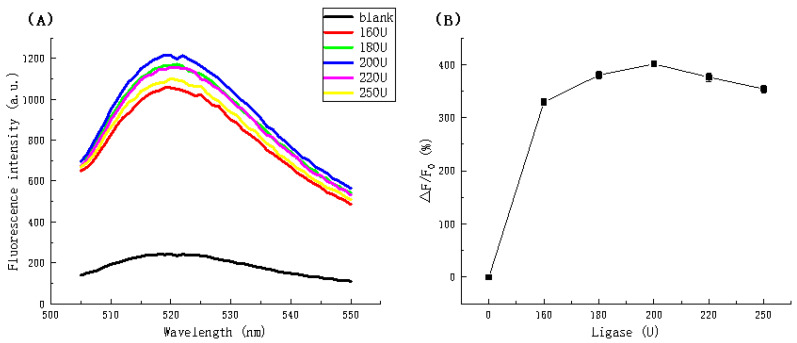
(**A**) Fluorescence spectra and (**B**) Fluorescence intensity ratio (△F/F_0_) in the presence of different ligase dosages.

The fluorescence spectra in Figure 4A shows a gradual increase in fluorescence intensity up to a maximum (1213.365 a.u.) dosage of 200 U followed by a reduction in intensity at concentration higher than 200 U. Figure 4B also indicates the same trend, clearly indicating a dosage of 200 U is optimal.

#### 3.3.3. DNA Polymerase

The polymerase has unique chain replacement and continuous synthesis properties and is able to continuously synthesize DNA fragments as long as 70 kb. In the RCA reaction, the phi29 DNA polymerase plays a key role. In the presence of dNTPs and phi29 polymerase buffer, the closed loop synthesizes long-stranded DNA numerous repeating units. The polymerase is active in mild conditions; thus, the amplification of short-stranded DNA to long-stranded DNA is possible at room temperature.

**Figure 5:**
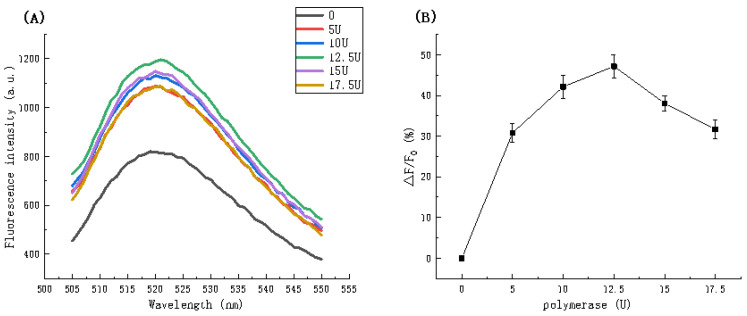
(**A**) Fluorescence spectra in the presence of different polymerase dosages. (**B**) The relationship between fluorescence intensity ratio (△F/F_0_) and polymerase dosage.

Figure 5 shows the relationship between fluorescence intensity and polymerase dosage. Figure 5A indicates an initially increasing trend followed by a decrease. Among the dosages analyzed, 12.5 U resulted in the highest fluorescent intensity (1204.544 a.u.) for the polymerization system. Figure 5B illustrates the relationship more clearly. At dosages between 0 and 10 U, the fluorescence intensity ratio gradually increases and between 12.5 U and 15 U, the intensity clearly diminishes. It is apparent that a dosage of 12.5 U is optimal.

#### 3.3.4. dNTPs Concentration

The dNTP mixture contains four deoxynucleotides (dATP, dCTP, dGTP and dTTP), with each nucleotide able to assemble into long chains with a specific sequence during DNA polymerization. In the presence of DNA polymerase, primer DNA, polymerase buffer and dNTPs, the reaction system begins at an increased pace and eventually forms a long-stranded chain. Thus, demonstrating that the dNTPs influence the outcome of the reaction.

**Figure 6:**
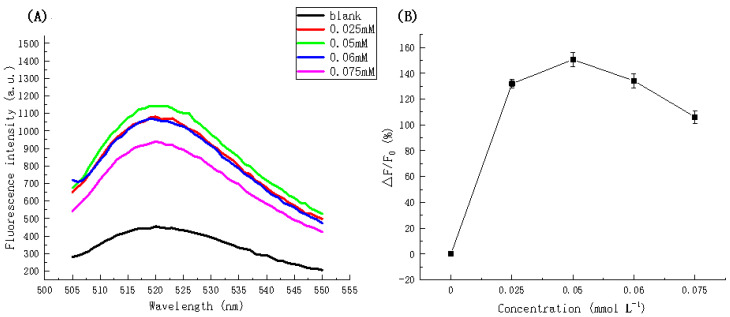
(**A**) Fluorescence spectra in the presence of different concentrations of dNTPs. (**B**) Fluorescence intensity ratio (△F/F_0_) at an emission wavelength of 520 nm in the presence of different concentrations of dNTPs.

Figure 6 illustrates the fluorescence intensity with respect to dNTP concentration. The fluorescence intensity at a concentration of 0.05 mmol L^−^^1^ corresponds to the maximum value (1148.926 a.u.) and the fluorescence intensity of the other concentrations is lower than that for 0.05 mmol L^−^^1^. This relationship is more clearly illustrated in Figure 6B where the fluorescence intensity ratio at an emission wavelength of 520 nm in the presence of different dNTPs concentrations is shown. Hence, a concentration of 0.05 mmol L^−^^1^ was selected as the optimal concentration.

#### 3.3.5. H1 and H2 Concentration

H1 and H2 are single-stranded DNA fragments composed of different sequences. The base sequences of H1 and H2 are composed of three parts: the substrate binding region, the catalytic core region and the assembly promoting region. In this study, following completion of the RCA reaction, H1 and H2 were added. The two single-stranded assembly-promoting regions (H1 and H2) and the amplified long single-strand hybridized to successfully form a deoxyribozyme with a catalytic core region. Once the addition of the substrate occurs, the substrate binding area and the substrate hybridize and the substrate cleavage reaction occurs under the action of Mg^2+^, thereby separating the fluorescent group from the quenching group and resulting in the generation of a fluorescent signal. It follows that the concentration of H1 and H2 has a substantial influence on fluorescence intensity.

**Figure 7:**
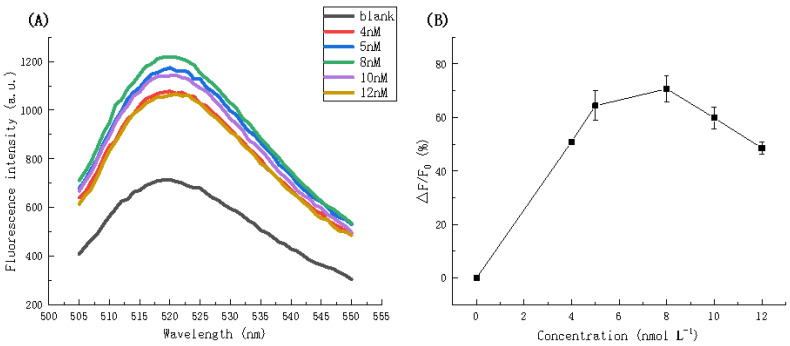
(**A**) Fluorescence spectra in the presence of different concentrations of H1 and H2. (**B**) Fluorescence intensity ratio (△F/F_0_) at an emission wavelength of 520 nm in the presence of different concentrations of H1 and H2.

Figure 7A shows the fluorescence spectra obtained at different H1 and H2 concentrations and Figure 7B shows the fluorescence intensity ratio of the system with different H1 and H2 concentrations at an emission wavelength of 520 nm. It is clear that the maximum fluorescence intensity (1216.699 a.u.) is achieved at H1 and H2 concentrations of 8 nmol L^−^^1^; therefore, a concentration of 8 nmol L^−^^1^ was used for H1 and H2 in subsequent experiments.

#### 3.3.6. Mg^2+^ Concentration

When present at physiological concentrations within cells, Mg^2+^ is an important factor in maintaining genome stability. It stabilizes the structure of DNA and chromatin and is an essential cofactor for the enzymatic system supporting DNA synthesis and decomposition. It is also used as a cofactor during nucleoside excision repair, base excision repair and mismatch repair. In this study, Mg^2+^ promoted the assembly of DNAzyme and drove substrate cleavage reactions. When the cleavage reaction was complete, the originally quenched fluorophore regained its fluorescent properties; hence, it is an important component in the process of fluorescence intensity changes within this reaction system.

**Figure 8:**
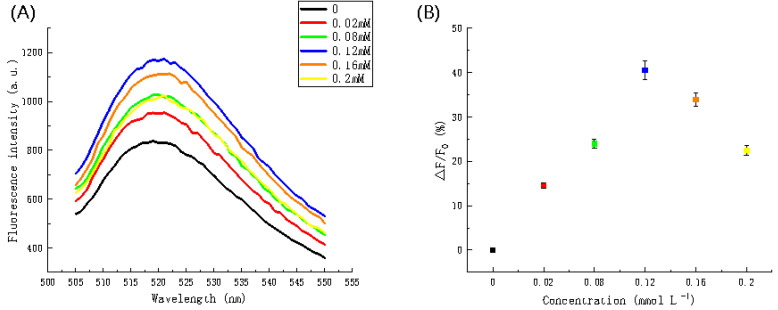
The impact of Mg^2+^ concentration on fluorescence intensity. (**A**) Fluorescence spectra and (**B**) scatter plot of Mg^2+^ concentration versus fluorescence intensity.

Figure 8 showed the relationship between fluorescence intensity and Mg^2+^ concentration. In Figure 8A, the fluorescence intensity reached a maximum (1179.511 a.u.) corresponding to a concentration of 0.12 mmol L^−^^1^. Figure 8B showed a scatter plot that illustrates the same trend more distinctly. At concentrations between 0 and 0.12 mmol L^−^^1^, the rate of changed gradually increases and then gradually decreased at concentrations higher than 0.12 mmol L^−^^1^. Thus, the selection of a concentration of 0.12 mmol L^−^^1^ is reasonable.

#### 3.3.7. Substrate Concentration

With the addition of magnesium ions and the substrate, the cleavage deoxyribozyme was activated and the substrate cleavage reaction began. Since the two ends of the substrate were respectively labeled with a fluorescent group and a quenching group, the fluorescence intensity of the system should be low when it was not cut. The cleavage reaction would cause the fluorescent group and the quenching group to separate and, finally, fluorescence recovered.

**Figure 9:**
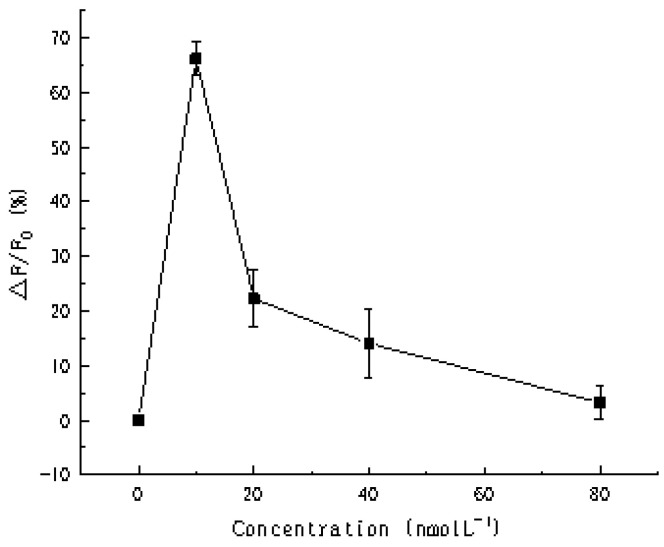
Fluorescence intensity ratio (△F/F_0_) at an emission wavelength of 520 nm in the presence of different concentrations of substrate.

Figure 9 showed the fluorescence intensity ratio at different concentration of substrate, the △F/F_0_ reached the maximum when the substrate was 10 nmol L^−1^. Thus, it is reasonable to choose this concentration to conduct follow-up experiments.

#### 3.3.8. Sensor Selectivity

Biosensors generally require high selectivity. In this study, miRNA-16 (similar to miRNA-21) and mismatch miRNA-21, which differs by only one base pair, were selected. As the combination of miRNA and padlock probe occured during the initial stage of the reaction, theoretically, in the absence of miRNA-21 or miRNA with a base pair sequence mismatch, the ligation reaction will not occur and the subsequent RCA will not proceed. Therefore, the fluorescence intensity of miRNAs with different base pair sequences existed great differences.

**Figure 10:**
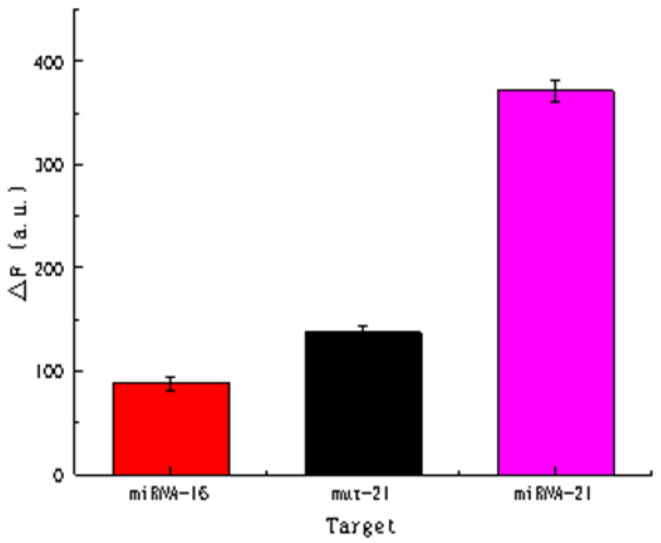
Selective detection of miRNA using the sensing system. The concentration of miRNA-16 and mut-miRNA was 5 nmol L^−1^, while the concentration of miRNA-21 was 5 pmol L^−1^.

From the information shown in Figure 10, it can be seen that fluorescence intensity signals for the target miRNA-21 detected at a concentration of 5 pmol L^−1^ were substantially higher than those for miRNA-16 and mut-miRNA-21 at concentrations of 5 nmol L^−1^. This indicated that the sensor is highly selective.

#### 3.3.9. Analytical Performance of the Sensing System for miRNA-21 Detection

**Figure 11:**
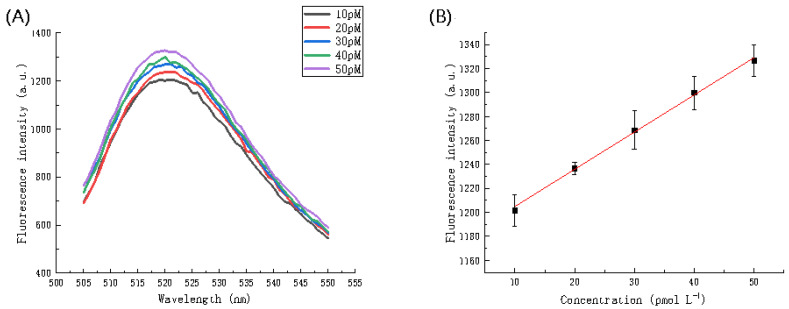
(**A**) Fluorescence spectra in the presence of different concentrations of miRNA (in buffer). (**B**) The relationship between fluorescence intensity in the buffer solution and the miRNA concentration. The error bars represent the standard deviation of three repeat experiments.

The purpose of this study was to detect miRNA-21; therefore, following completion of the above optimization experiments the detection of different concentrations of miRNA-21 under the optimized conditions was performed to determine the sensitivity and quantitative detection performance of the sensor. Figure 11A showed that as the concentration of miRNA increases, the fluorescence intensity gradually increases. Figure 11B showed a linear relationship between miRNA concentration and fluorescence intensity. As the miRNA concentration increased from 10 pmol L^−1^ to 50 pmol L^−1^, the fluorescence intensity also gradually increased. Figure 11B served as the calibration curve for concentration and fluorescence intensity. The calibration curve indicated a linear relationship between 10 pmol L^−1^ and 50 pmol L^−1^. The linear regression equation is F = 1173.9566 + 3.10523C (R^2^ = 0.9972), where F and C represent the fluorescence intensity at a wavelength of 520 nm and miRNA concentration (pmol L-1), respectively. The limit of detection (LOD) was calculated as 4 pmol L^−1^, estimated to be three times the blank (without miRNA-21) standard deviation divided by the slope. This LOD value indicates that the reported method may be applied for the detection of miRNA in amounts as low as 4 pmol L^−1^.

**Figure 12:**
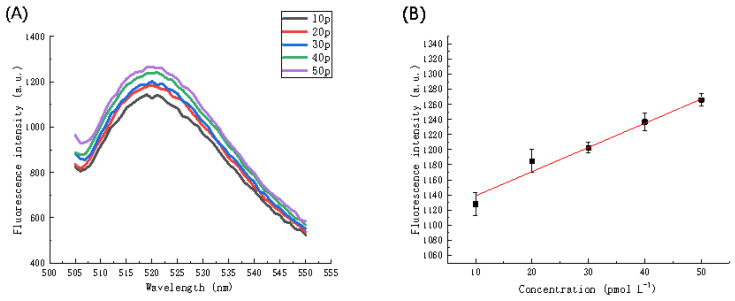
(**A**) Fluorescence spectra in the presence of different concentrations of miRNA (in serum). (**B**) Calibration curve of fluorescence intensity and miRNA concentration in serum. The error bars represent the standard deviation of three repeat experiments.

Table 2 shows that the LOD calculated using the proposed method is competitive with respect to similar strategies for the detection of miRNA based on RCA or DNAzymes. Next, 1% serum samples were spiked with different concentrations of miRNA and analyzed. The results in Figure 12A,B indicated that from concentrations of 10 pmol L^−1^ to 50 pmol L^−1^, the fluorescence intensity gradually increased. A linear regression analysis of the fluorescence intensity at different concentrations of miRNA using a wavelength of 520 nm was performed to obtain a calibration curve of concentration and fluorescence intensity. The linear regression equation is F = 1106.88387 + 3.20306C (R^2^ = 0.97712), the LOD in serum is 6.255 pmol L^−1^, which is similar to the LOD value obtained for miRNA in the buffer solution. Therefore, it is possible to quantitatively analyze the target miRNA in the concentration range 10–50 pmol L^−1^.

## 4. Conclusions

In conclusion, a novel, fluorescent miRNA biosensor is presented. The operating principle of the miRNA biosensor is based on RCA and MNAzyme dual signal amplification. This sensing strategy offers a simple and highly sensitive method with a LOD as low as 4 pmol L^−1^. Furthermore, the biosensor is extremely selective and is able to distinguish between miRNAs with one base pair mismatch. Therefore, the proposed biosensor offers a novel and effective strategy for the detection of miRNA using a combination of RCA and DNAzymes.

## Figures and Tables

**Table 1:** Sequences of the oligonucleotides used in this study.

Name	Sequence(5’-3’)
Padlock probe	pCTGATAAGCTACGAATGGCGTTATGCCTCAATTAGAAGTCTTATGCGAAAGCGTGACGGCTAATGGACTGCAGTCAACATCAGT
MiR-21	UAG CUU AUC AGA CUG AUG UUG A
MiR-16	UAG CAG CAC GUA AAU AUU GGC G
Mut-miR-21	UAG CUU AAC AGA CUG AUG UUG A
H1	TC AAT TAG A AAG CAC CCA TGT TAC TCT
H2	GAT ATC AGC GAT CTT AG TCT TATG
Substrate	BHQ-1-AGA GTA TrAG GAT ATC-FAM

**Table 2:** Comparison of the proposed method with similar strategies for the detection of miRNA based on RCA or DNAzymes.

Detection Strategy	LOD	R^2^	Reference
RNA-cleaving DNAzymes	0.2 nM	0.996	[32]
RCA and CHA	87 fM	0.9908	[24]
Autonomous catalytic assembly of DNAzymes	10 pM	0.984	[33]
Target-primed and branched RCA	10 fM	0.9994	[34]
Peroxidase-mimicking system composed of trimeric G-triplex and hemin DNAzyme	37 fM	0.999	[35]
RCA and triple-helix molecular switch-actuation	1.1 aM	0.9997	[36]
Exonuclease III-propelled integrated DNAzyme	100 fM	0.998	[37]
RCA and multi-component nucleic acid enzymes	4 pM	0.9972	This work

## Data Availability

Not applicable.
